# Intracellular enhancement technique for gadoxetic acid-enhanced hepatobiliary-phase magnetic resonance imaging: evaluation of hepatic function

**DOI:** 10.1007/s00261-025-04817-y

**Published:** 2025-01-31

**Authors:** Dara Fonseca, Yuko Nakamura, Toru Higaki, Shogo Maeda, Takashi Nishihara, Yoshitaka Bito, Masahiro Takizawa, Shota Kondo, Ryo Higashino, Shintaro Morishita, Yuji Akiyama, Shingo Fukuma, Tomokazu Kawaoka, Masataka Tsuge, Shiro Oka, Kazuo Awai

**Affiliations:** 1 Hiroshima University, Hiroshima City, Japan; 2 FUJIFILM Corporation, Shintoyofuta, Kashiwa City, Japan; 3 Hokkaido University Graduate School of Medicine, Sapporo City, Japan; 4 Hiroshima University Hospital, Hiroshima City, Japan

**Keywords:** Intracellular enhancement technique, Motion-sensitized driven equilibrium pulse, Gadoxetic acid, Magnetic resonance imaging, Hepatic function

## Abstract

**Purpose:**

To investigate the utility of intracellular enhancement (ICE) technique which suppresses signals from the extracellular space for the evaluation of hepatic function on gadoxetic acid-enhanced hepatobiliary-phase (HBP) images.

**Methods:**

We subjected 67 patients with suspected neoplastic hepatic lesions to gadoxetic acid-enhanced HBP imaging with and without ICE [i-HBP, conventional-HBP (c-HBP)]. A radiologist calculated the liver/spleen contrast (LSC) [LSC = signal intensity (SI) of liver/SI of spleen]. Receiver-operating analysis was used to evaluate the diagnostic value of the LSC on i-HBP- (i-LSC) and c-HBP images (c-LSC) to differentiate between Child-Pugh classes A and B.

**Results:**

Of the 67 patients, 57 were in Child-Pugh class A and 10 were in class B. For their differentiation, the area under the curve value of i-LSC was higher than of c-LSC (0.81 vs. 0.68).

**Conclusions:**

ICE technique can improve the accuracy of estimating hepatic function on HBP images.

**Graphical abstract:**

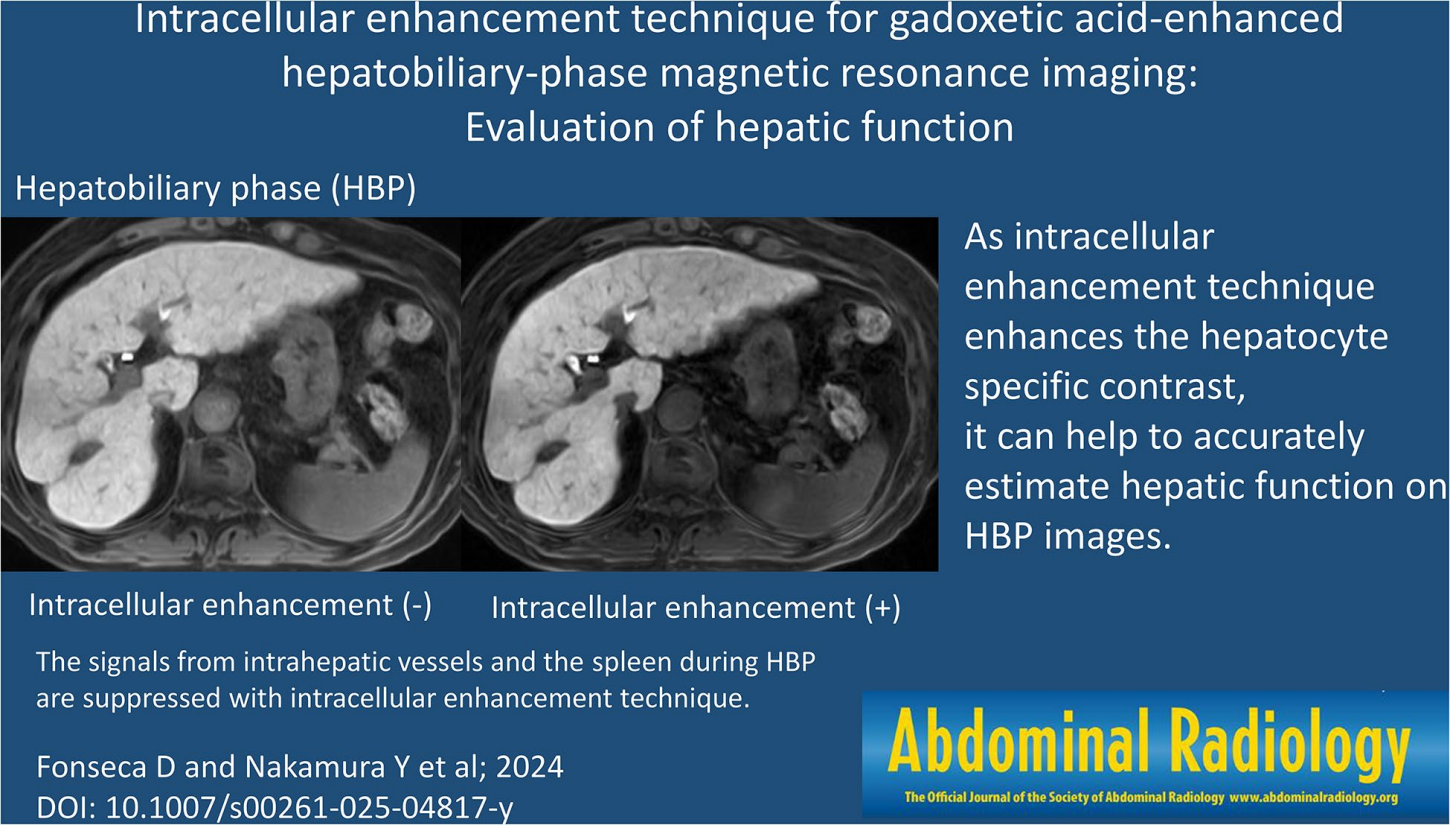

**Supplementary Information:**

The online version contains supplementary material available at 10.1007/s00261-025-04817-y.

## Introduction

The liver function must be known to predict the clinical outcomes and prevent postoperative hepatic failure in patients with liver disease or cirrhosis. To assess their liver function reserve, biochemical parameters, clinical scoring systems, and the pre-treatment liver volume are evaluated [1–3]. The indocyanine-green clearance test helps to comprehensively assess the liver function reserve [4]. However, such methods reflect only the total- but not the regional liver function.

As gadoxetic acid-enhanced MRI yields dynamic- and hepatobiliary-phase (HBP) information, it helps to detect and characterize focal hepatic lesions [5–9]. Because of its lipophilic characteristics, gadoxetic acid is absorbed by functional hepatocytes and secreted into the biliary system without changing its chemical structure [10]. Consequently, hepatic enhancement during HBP is valuable for the quantitative assessment of liver function [11]. However, as gadoxetic acid acts as both an extracellular- and hepatocyte-specific contrast agent, simple signal measurements may be affected not only by hepatocyte specific- but also by extracellular-space enhancement, resulting in the inaccurate estimation of liver function [3]. A more accurate estimation would be obtained when only hepatocyte-specific enhancement is depicted.

To obtain only hepatocyte-specific enhancement, an intracellular enhancement (ICE) technique that suppresses signals from the extracellular space using a motion-sensitized driven equilibrium was developed [12]. To investigate the utility of this technique for the evaluation of hepatic function we compared HBP images acquired with and without the ICE technique.

## Materials and methods

### Patients

This prospective study was approved by the Human Ethics Review Committee of our institute and signed informed consent was obtained from all study subjects. Patient records and information were anonymized and de-identified prior to analysis.

Consecutive patients who underwent clinically indicated gadoxetic acid-enhanced MRI at our institution were enrolled between February 2023 and January 2024. The exclusion criteria were as follows: (1) pediatric patients; (2) patients who failed to achieve all HBP scanning protocols; (3) contraindications for MRI (e.g., cardiac pacemakers or metal implants).

Using the hospital information system we obtained patient data recorded within 30 days of their MRI studies. They included the patient gender and age, the serum albumin-, asparate aminotransferase (AST), alanine aminotransferase (ALT), total bilirubin, platelet levels, the prothrombin time-international normalized ratio (PT–INR), and the Child-Pugh score.

### MRI protocol

#### Intracellular enhancement technique

The motion-sensitized driven equilibrium (MSDE) pulse suppresses signals from the blood flow by adding motion-proving gradients (MPGs) with a low b-value [13, 14]. Thus it increases hepatocyte-specific enhancement during HBP by suppressing signals from the blood flow in the extracellular space. The ICE technique combines MSDE and fat-saturated T1-weighted gradient-echo nature of the sequence (TIGRE) to increase hepatocyte-specific contrast enhancement on HBP images [12]. Briefly, the MSDE pulse is composed of three composite radiofrequency hard pulses, MPGs, and spoiler gradients. The fat saturation pulse is comprised of chemical shift-selective pulses and spoiler gradients. The MSDE pulse (b-value 60 s/mm^2^, duration 21 ms) is applied immediately before the fat saturation pulse (duration 100 ms). The 3D RF-spoiled gradient-echo sequence is applied after the MSDE pulse and the fat saturation pulses to acquire T1-weighted signals modified by the preparation pulses.

### Image acquisition

Scanning was on a 3T MRI instrument (FUJIFILM Corporation, Tokyo, Japan) using a 28-channel coil. Twenty-five µmol/kg of gadoxetic acid (EOB-Primovist, Bayer Yakuhin, Osaka, Japan), was injected intravenously at a rate of 2.0 mL/s followed by 20 mL of saline delivered at the same rate using a power injector (Sonic Shot 50; Nemoto-Kyorindo, Tokyo, Japan). HBP imaging was started 20 min after the contrast injection. Imaging was with TIGRE with parallel imaging (rapid acquisition through a parallel imaging design; RAPID, FUJIFILM Corporation).

Diffusion-weighted images of the liver feature low resolution, noise, and artifacts [15]. As the MSDE pulse used in the ICE technique suppresses signals from the blood flow by adding motion-proving gradients with a low b-value [13, 14], ICE may degrade the image quality due to the MSDE pulse. Wavelet denoising with geometry factor weighting (g-denoising) can reduce the image noise by adapting to spatially varying noise levels induced by parallel imaging; it yields better image quality than conventional HBP images [16]. Thus, g-denoising was added to our ICE protocol for HBP scanning.

As ICE and g-denoising may affect the contrast on HBP images acquired with our ICE scanning protocol, we scanned them using 3 protocols to examine the effect of ICE and g-denoising separately. The imaging protocols involved conventional HBP- (c-HBP), HBP with g-denoising- (g-HBP), and HBP with g-denoising and ICE (i-HBP) scanning.

The scan parameters for HBP were section thickness and interval 4.0 mm, TR/TE 4.0 msec/1.8 msec, flip angle 15°, field of view 36 cm, matrix 320 × 224, and parallel imaging factor 2.2 (2.0 for phase direction and 1.1 for slice direction). Parallel imaging reconstruction was performed in the k-space domain for c-HBP and in the image space domain for g-HBP and i-HBP scans because g-denoising can be applied to parallel imaging reconstruction performed in the image- but not in the k-space domain [16]. Although dynamic gadoxetic acid-enhanced MRI scans were obtained in the clinical studies they were not evaluated.

### Image analysis

Quantitative analysis was performed by a radiologist (DF with 3 years of radiology experience) and a board-certified radiologist (SM with 7 years of radiology experience). The signal intensity (SI) of the hepatic parenchyma was recorded as the mean measurement value of 4 region of interests (ROIs) in the right anterior-, right posterior-, left medial-, and left lateral hepatic segment. Areas of focal changes in hepatic parenchyma, in large vessels, and prominent artifacts were carefully avoided. The SI of the spleen was recorded on the level of the largest spleen area. Each value was calculated by averaging 3 measurements.

The 2 radiologists calculated the liver/spleen contrast (LSC = ROI_liver_/ ROI_spleen_, where ROI_liver_ is the mean SI of the hepatic parenchyma and ROI_spleen_ the mean SI of the spleen [11]. Each value recorded by the two radiologists was averaged. We defined LSC at c-HBP as c-LSC, LSC at g-HBP as g-LSC, and LSC at i-HBP as i-LSC.

### Statistical analysis

All statistical analyses were with JMP17 software (SAS Institute, Cary, NC). Differences were determined with the Fisher test for the patient gender and the Mann-Whitney *U*-test for the other parameters. Each LSC value was subjected to receiver-operating characteristic (ROC) analysis to determine the threshold value yielding the highest sensitivity and specificity to differentiate between Child-Pugh classes A and B. Differences of *p* < 0.05 were considered statistically significant. For multiple comparisons differences of *p* < 0.017 using Bonferroni correction were considered statistically significant.

## Results

### Patient background factors

**Fig. 1:**
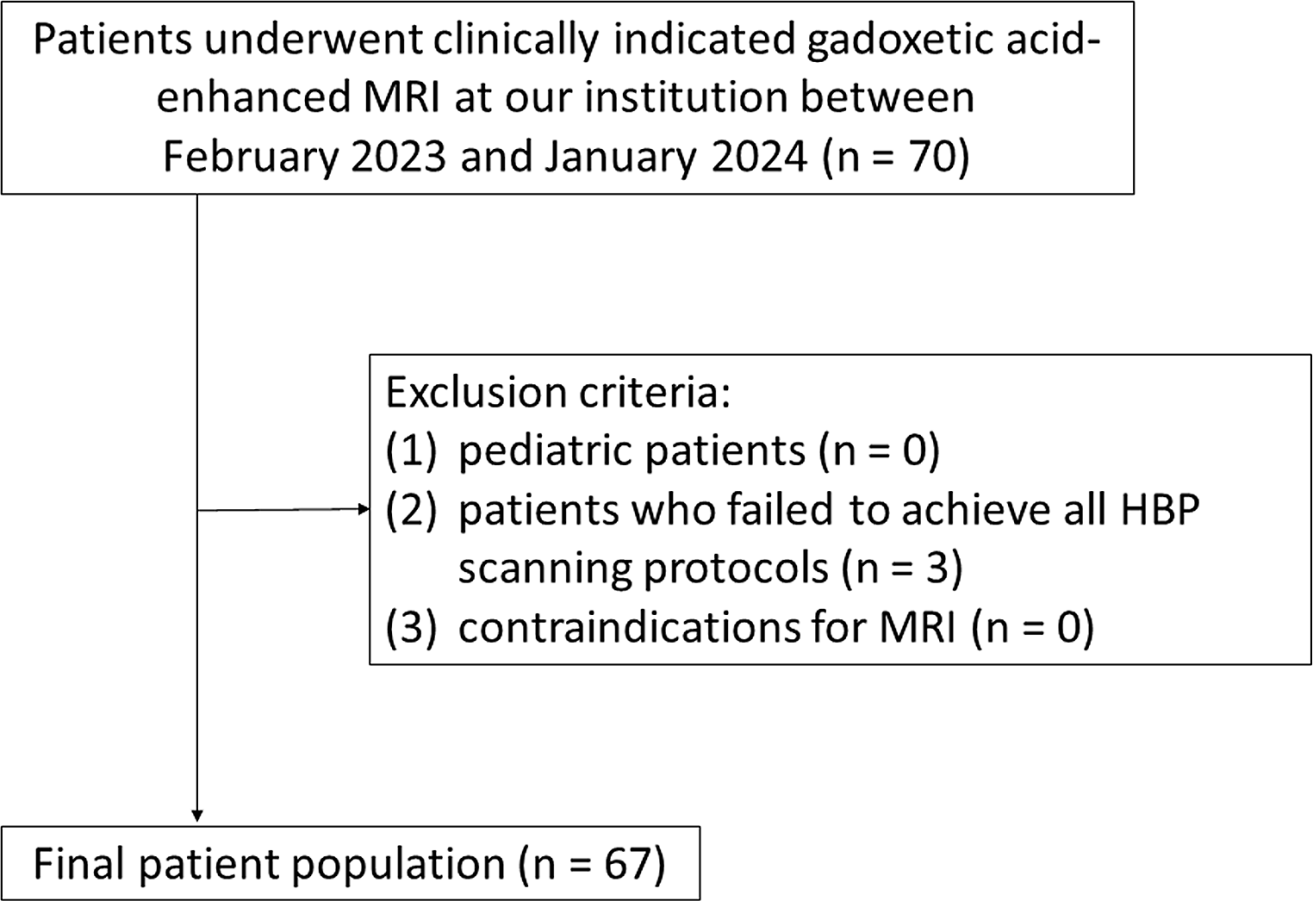
Patient flowchart

A total of 67 patients (45 men, 22 women; age range, 22–88 years; median age, 74.0 years) were enrolled (Fig. 1). Of these 42 were followed up after malignant liver tumor surgery, 5 underwent MRI to assess liver lesions detected on ultrasound- or dynamic CT studies, 14 were screened for liver tumors, and 2 each were followed for benign liver tumors, required staging of a suspected malignant liver tumor, or were assessed for the effect of liver tumor therapy.

Of the 67 patients, 57 were in Child-Pugh class A; 10 were in class B. Table 1 shows patient characteristics. The serum albumin level and PT-INR were significantly lower in patients with Child-Pugh class B than class A (both *p* < 0.01). The patient age, sex, total bilirubin-, AST-, ALT-, and platelet levels were not significantly different (*p* = 0.05–0.72).

**Table 1:** Patient characteristics

	Child-Pugh class A	Child-Pugh class B	*p* value
Number of patients*	57	10	
Age (years)	74 (22–88)	70.5 (37–81)	0.35
Sex (male: female)*	39:18	6:4	0.72
Albumin (mg/dL)	4.1 (3.0–5.0)	3.4 (2.8–4.1)	< 0.01
Total bilirubin (mg/dL)	0.9 (0.3–2.4)	1.7 (0.3–5.2)	0.05
AST (IU/L)	24 (15–53)	27 (10–59)	0.16
ALT (IU/L)	19 (12–61)	18.5 (7–41)	0.62
PT-INR	90.5 (21–112)	71 (37–86)	< 0.01
Platelet count (x 10^3^/µL)	168 (18–414)	239.5 (51–350)	0.32

Note. – Unless otherwise indicated, data are the median (range)

* Data are number of patients

AST, aspartate aminotransferase; ALT, alanine aminotransferase; PT-INR, prothrombin time-international normalized ratio

### Image analysis

**Fig. 2:**
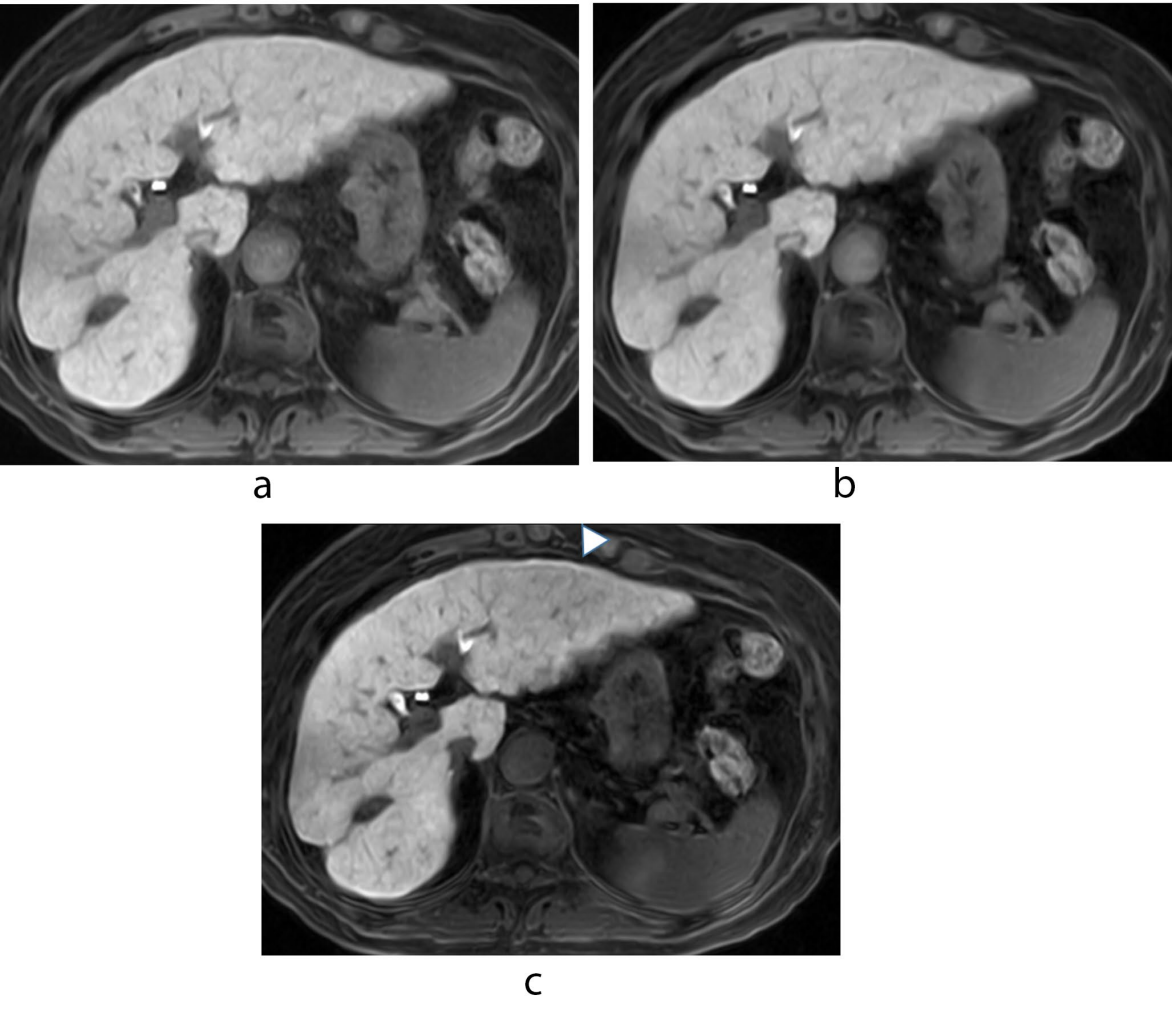
A 74-year-old woman with Child-Pugh class A. (**a**) Conventional hepatobiliary-phase (HBP) image (**b**) HBP image with g-denoising (**c**) HBP image with g-denoising and the intracellular enhancement technique. On (**c**) the signals from intrahepatic vessels and the spleen are suppressed and the degree of enhancement of the hepatic parenchyma is slightly better than on the images presented in (**a**) and (**b**). The slight artifact due to the motion-sensitized driven equilibrium (MSDE) pulse (arrowhead) is confirmed on image (**c**). The liver/spleen contrast (LSC) was 1.76, 1.78, and 2.77 for (**a**), (**b**), and (**c**), respectively

**Fig. 3:**
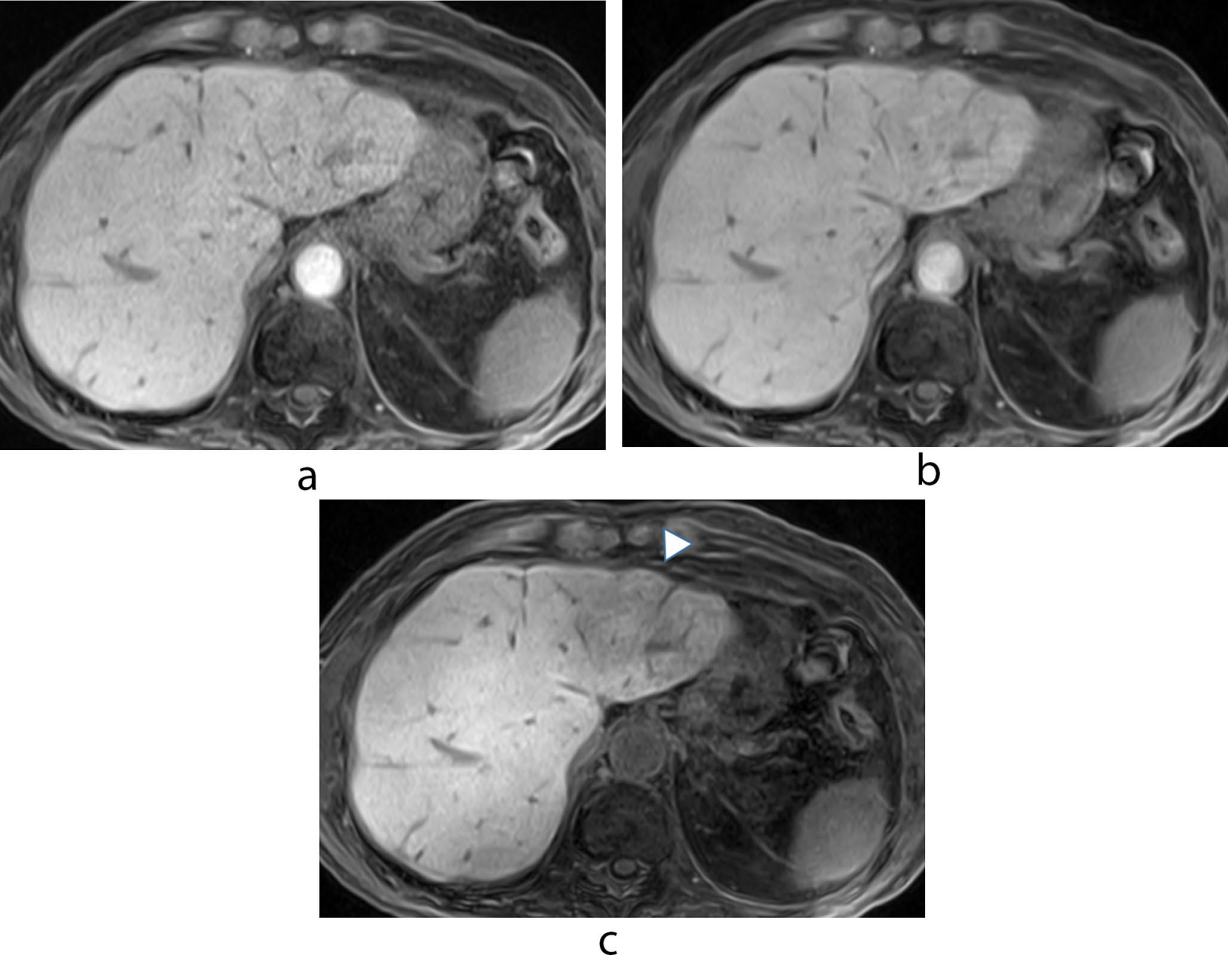
A 79-year-old man with Child-Pugh class B. (**a**) Conventional hepatobiliary phase (HBP) image (**b**) HBP image with g-denoising (**c**) HBP image with g-denoising and the intracellular enhancement technique. The signals from intrahepatic vessels and the spleen and the degree of hepatic parenchymal enhancement are very similar on images (**a**), (**b**), and (**c**). As shown in (**c**), the artifact attributable to the motion-sensitized driven equilibrium (MSDE) pulse is prominent especially in the left hepatic lobe (arrowhead). The liver/spleen contrast (LSC) was 1.12, 1.17, and 1.96 for (**a**), (**b**), and (**c**), respectively

Table 2 shows the LSC obtained with our 3 protocols. In all 67 patients i-LSC was significantly higher than c-LSC and g-LSC (both *p* < 0.01); there was no significant difference between c-LSC and g-LSC (*p* = 0.19). In patients with Child-Pugh class A, i-LSC was significantly higher than c-LSC and g-LSC (both *p* < 0.01); there was no significant difference between c-LSC and g-LSC (*p* = 0.21). In patients with Child-Pugh class B there were no significant differences under the 3 scanning protocols (*p* = 0.72, 0.03, and 0.03 for c-LSC vs. g-LSC, c-LSC vs. i-LSC, and g-LSC vs. i-LSC, respectively) (Figs. 2 and 3, Supplementary Fig. 1).

**Table 2:** Liver/spleen contrast obtained with the 3 scanning protocols

	c-LSC	g-LSC	i-LSC	*p* value		
				c-LSC vs. g-LSC	c-LSC vs. i-LSC	g-LSC vs. i-LSC
All (*n* = 67)	1.57 (0.93–2.40)	1.56 (0.90–2.54)	2.25 (0.97–5.23)	0.19	< 0.01	< 0.01
Child-Pugh class A (*n* = 57)	1.59 (0.93–2.40)	1.59 (0.90–2.54)	2.30 (1.13–5.23)	0.21	< 0.01	< 0.01
Child-Pugh class B (*n* = 10)	1.42 (1.01–1.77)	1.35 (0.97–1.90)	1.55 (0.97–2.45)	0.72	0.03	0.03

Data are the median (range)

c-LSC: liver/spleen contrast at conventional hepatobiliary-phase (HBP) scanning

g-LSC: liver/spleen contrast at HBP scanning with g-denoising

i-LSC: liver/spleen contrast at HBP scanning with g-denoising and intracellular enhancement technique

Table 3 lists the LSC obtained with our 3 scanning protocols for patients with Child-Pugh class A and B. It was significantly higher in patients with Child-Pugh class A than B at i-HBP (*p* < 0.01), but not both at c-HBP and g-HBP (*p* = 0.08 and 0.05, respectively) (Supplementary Fig. 2).

**Table 3:** Comparison of the liver/spleen contrast obtained with the 3 scanning protocols to differentiate between child-pugh class A and B

Protocol	Child–Pugh class A (*n* = 57)	Child–Pugh class B (*n* = 10)	*p* value
c-LSC	1.59 (0.93–2.40)	1.42 (1.01–1.77)	0.08
g-LSC	1.59 (0.90–2.54)	1.35 (0.97–1.90)	0.05
i-LSC	2.30 (1.13–5.23)	1.55 (0.97–2.45)	< 0.01

Data are the median (range)

c-LSC: liver/spleen contrast at conventional hepatobiliary-phase (HBP) scanning

g-LSC: liver/spleen contrast at HBP scanning with g-denoising

i-LSC: liver/spleen contrast at HBP scanning with g-denoising and intracellular enhancement technique

**Fig. 4:**
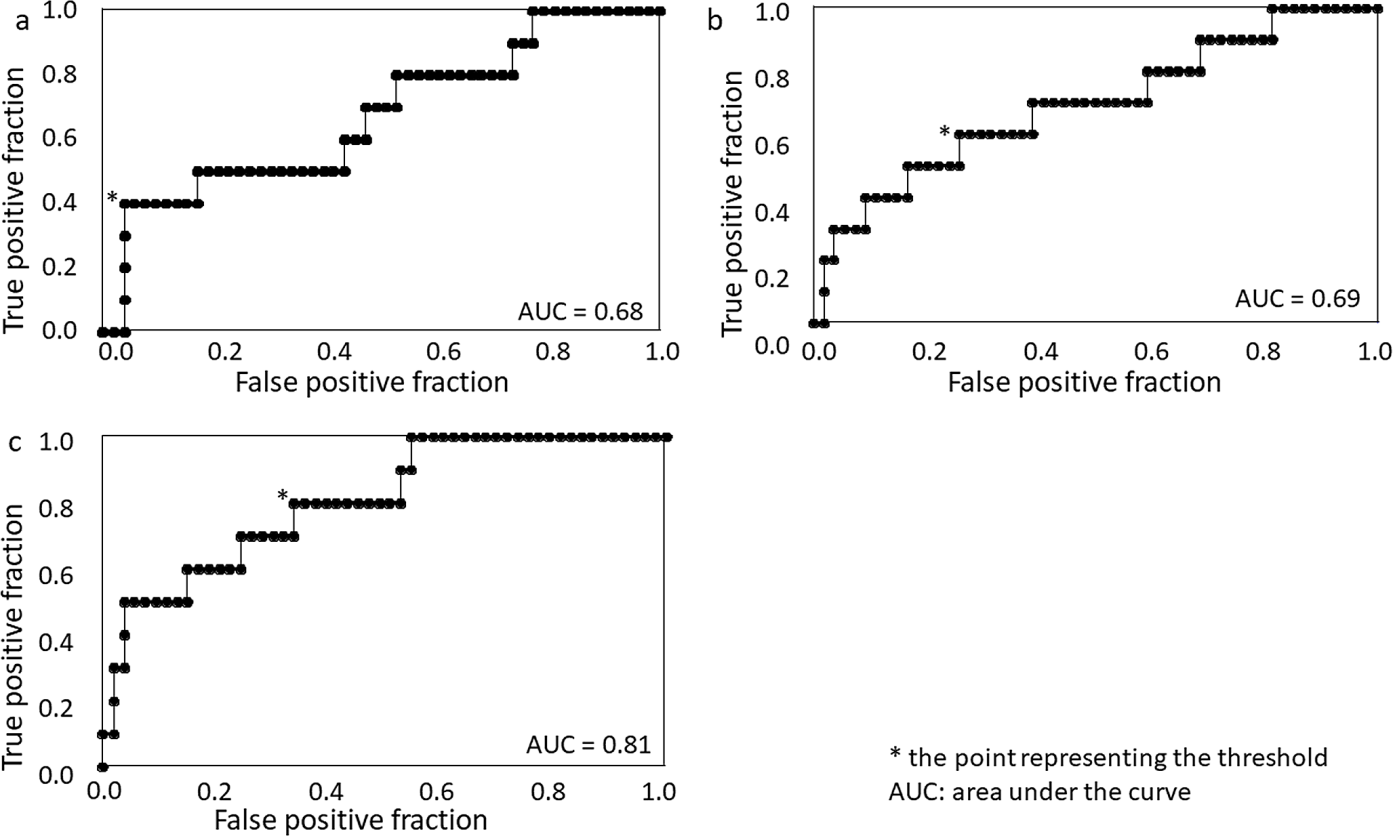
Receiver-operating characteristic curve analyses of the liver/spleen contrast (LSC) on HBP scans. (**a**) c-LSC (**b**) g-LSC (**c**) i-LSC. c-LSC, LSC at conventional hepatobiliary phase (HBP); g-LSC, LSC at HBP with g-denoising; i-LSC, LSC at HBP with g-denoising and the intracellular enhancement technique

For the differentiation between Child-Pugh class A and B, the value of i-LSC [area under the curve (AUC) 0.81; cutoff 2.12, sensitivity 80.0%, specificity 66.7%] was higher than of c-LSC (AUC 0.68; cutoff 1.12, sensitivity 40.0%, specificity 96.3%) and g-LSC (AUC 0.69; cutoff 1.43, sensitivity 60.0%, specificity 74.1%) (Fig. 4).

## Discussion

With our 3 protocols, i-LSC was significantly higher than c-LSC and g-LSC. In patients with Child-Pugh class A, i-LSC was significantly higher than c-LSC and g-LSC while there were no significant differences in patients with Child-Pugh class B. The higher the liver function, the more gadoxetic acid is taken up by hepatocytes, revealing that ICE increased LSC by enhancing the hepatocyte-specific contrast. The LSC was higher in patients with Child-Pugh class A than B only on i-HBP scans and by ROC analysis their AUC value was highest among the 3 scanning methods. Based on our findings we concluded that ICE can improve the accuracy of hepatic function estimation on HBP scans.

As ICE may degrade the image quality due to the MSDE pulse, we applied g-denoising to the i-HBP scans. We found that while there was no significant difference on the c-LSC and the g-LSC, the i-LSC was significantly higher than c-LSC. The AUC value of i-LSC- was greater than of c-LSC; it was very similar on c-LSC and g-LSC. We suggest that the ICE technique, but not g-denoising, helps to accurately estimate hepatic function on i-HBP scans.

The only noise reduction technique available on the scanner we used is g-denoising. It can reduce the image noise by adapting to spatially varying noise levels induced by parallel imaging [16]. We used it to improve the image quality of HBP scans using ICE. However, g-denoising failed to completely eliminate artifacts due to the MSDE pulse (Figs. 2 and 3). To improve ICE, a technique that maximizes artifact reduction due to the MSDE pulse is needed. As an improved MSDE (iMSDE) pulse can mitigate the eddy currents as well as the inhomogeneity of B0 and B1 [17], it may reduce the artifact due to MSDE pulse. However, as iMSDE requires a longer duration or a lower b value than MSDE, the parameters should be optimized for iMSDE [12].

**Fig. 5:**
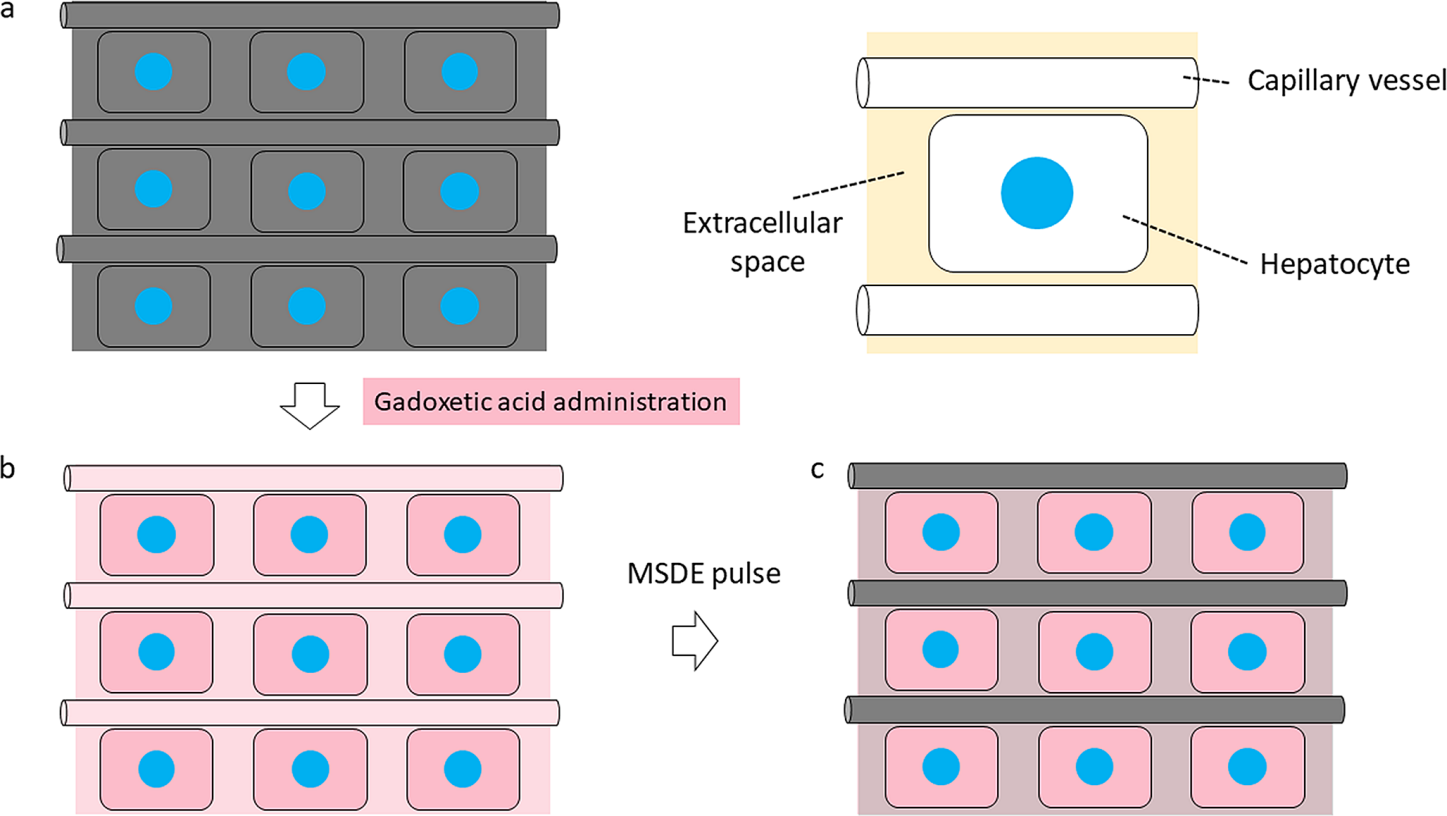
Schematic drawings of the effect of the intracellular enhancement technique on the hepatic parenchyma on hepatobiliary-phase images. (**a**) Before gadoxetic acid administration (pre-enhanced phase) (**b**) After gadoxetic acid administration (conventional hepatobiliary phase scanning) (**c**) After gadoxetic acid administration with applying the motion-sensitized driven equilibrium (MSDE) pulse (hepatobiliary phase scanning with the intracellular enhancement technique)

At a relatively low b-value (e.g., b < 200 s/mm^2^), signals decay rapidly because fast water movement in capillary vessels can cause substantial signal loss (perfusion-related diffusion) in addition to diffusion-induced signal attenuation (perfusion free diffusion). As the b-value increases, signals from the capillary vasculature are lost; this renders the diffusion process a dominant contributor to signal attenuation [18]. As an MSDE pulse with a b-value of 60 s/mm^2^ is applied, ICE should suppress signals from the capillary vasculature. Signals may also be slightly attenuated due to perfusion-free diffusion even at a relatively low b-value [18], indicating that ICE may slightly suppress such signals [12] (Fig. 5). Although we cannot confirm that the ICE technique suppresses signals from the extracellular- other than the intravascular space, our findings that i-HBP- but not c-HBP scanning improved the accuracy of hepatic function estimation suggest that the ICE technique is clinically useful.

The hepatocyte fraction, an index for the amount of hepatocyte uptake of gadoxetic acid referenced by splenic enhancement for the estimation of the extracellular space, is another technique to evaluate hepatocyte-specific enhancement. The hepatocyte fraction provides more information on liver function than simple signal measurements including the LSC [3]. However, the hepatocyte fraction assumes that enhancement of the extracellular space is the same as for the liver and spleen. Unlike hepatocyte fraction assessment, the application of ICE directly suppresses signals from the extracellular space using an MSDE pulse. This suggests that ICE reflects hepatocyte-specific enhancement more accurately than the hepatocyte fraction. Direct comparisons are needed to confirm the superior diagnostic value of the ICE technique.

We evaluated the LSC as an indicator of hepatic enhancement because the calculation method is simple and has been reported as a promising imaging biomarker for predicting the liver functional reserve [19, 11]. However, the signal intensity on MRI scans is affected by the scan parameters, the RF chain setup, and the field strength [20]. As enhancement of the splenic extracellular space increases with the deterioration of liver function [21, 22], the LSC value may not reflect hepatic enhancement accurately. T1 values, on the other hand, are only field- and tissue dependent. The diagnostic performance of the hepatic ΔT1 value, the difference of T1 value of hepatic parenchyma between pre- and post-gadoxetic acid, was superior to the LSC for differentiating between Child-Pugh class A- and class B [2, 23, 3]. Thus, direct comparison of the ICE technique with other advanced hepatic function imaging biomarkers including T1 mapping is required. In addition, the ICE technique should be evaluated using a method that can quantify hepatic enhancement more accurately. Further investigation is needed on these points.

Our study has some limitations. The study population was relatively small and our investigation was carried out at a single institution, meaning that our population may be biased. Indeed, the number of patients with Child-Pugh class B was small compared with Child-Pugh class A. In addition, the ICE technique is available only on the MRI scanner of one vendor currently. Therefore, we offer our findings as preliminary and studies involving large population and scanners of other vendors are needed in future. We used only the Child-Pugh classification for the evaluation of liver function because not all patients underwent the indocyanine green clearance test or pathological examination. Consequently, additional studies are needed to validate our findings. As ICE can improve hepatocyte-specific enhancement and can increase the contrast between non-hepatocyte lesions such as hepatic tumors and the surrounding hepatic parenchyma on HBP images, it raises tumor detectability. Although a 20 min delay for HBP scanning is widely accepted, it is too long and presents problems for throughput and raises costs in the clinical setting. ICE may shorten the delay time for HBP scanning and retain tumor detectability due to the improved contrast between tumors and the surrounding hepatic parenchyma. However, we did not investigate these issues and additional studies are needed to determine the diagnostic performance of i-HBP scanning.

## Conclusion

ICE improved the diagnostic performance of gadoxetic acid-enhanced HBP images for evaluating hepatic function based on Child-Pugh classification compared to the conventional scanning method. Based on our finding we suggest that the ICE technique can help to accurately estimate hepatic function on HBP images.

## Electronic supplementary material

Below is the link to the electronic supplementary material.

Supplementary Figure 1

Supplementary Figure 2

Supplementary Figure Legend

## Data Availability

No datasets were generated or analysed during the current study.

## Abbreviations

HBP: Hepatobiliary-phase
ICE: Intracellular enhancement
AST: Asparate aminotransferase
ALT: Alanine aminotransferase
PT-INR: Prothrombin time-international normalized ratio
MSDE: Motion-sensitized driven equilibrium
MPG: Motion-proving gradient
TIGRE: T1-weighted gradient-echo nature of the sequence
SI: Signal intensity
ROI: Region of interest
LSC: Liver/spleen contrast
ROC: Receiver-operating characteristic
AUC: Area under the curve
