# Supplementary figures and images for: Intracellular enhancement technique for gadoxetic acid-enhanced hepatobiliary-phase magnetic resonance imaging: evaluation of hepatic function
Source: Abdom Radiol (NY). 2025 Jan 31;50(8):3506–15. doi: 10.1007/s00261-025-04817-y (PMC12267321; doi:10.1007/s00261-025-04817-y)

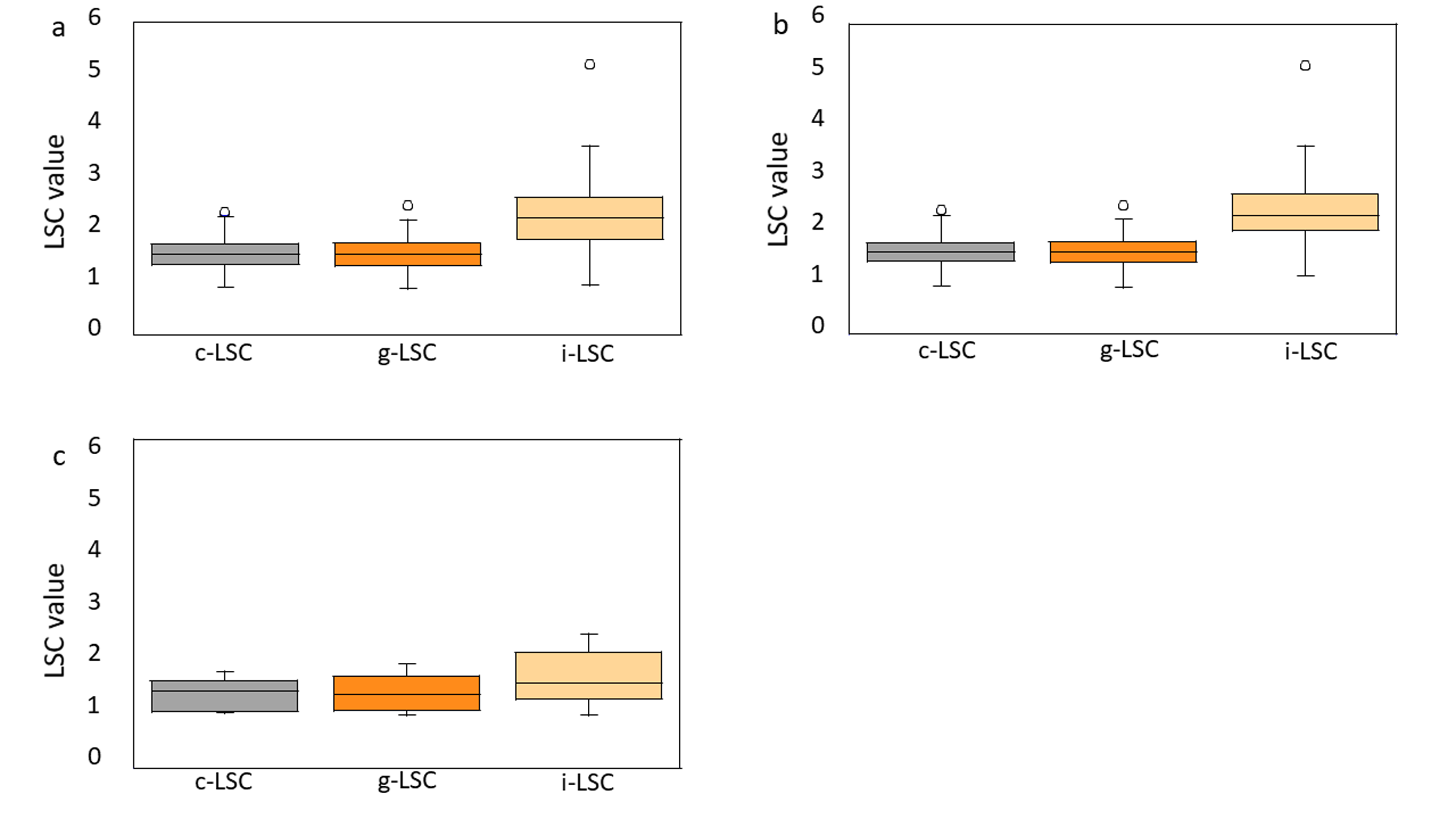

Supplement: Supplementary file 1 — Supplementary Figure 1 [file 261_2025_4817_MOESM1_ESM.tif]

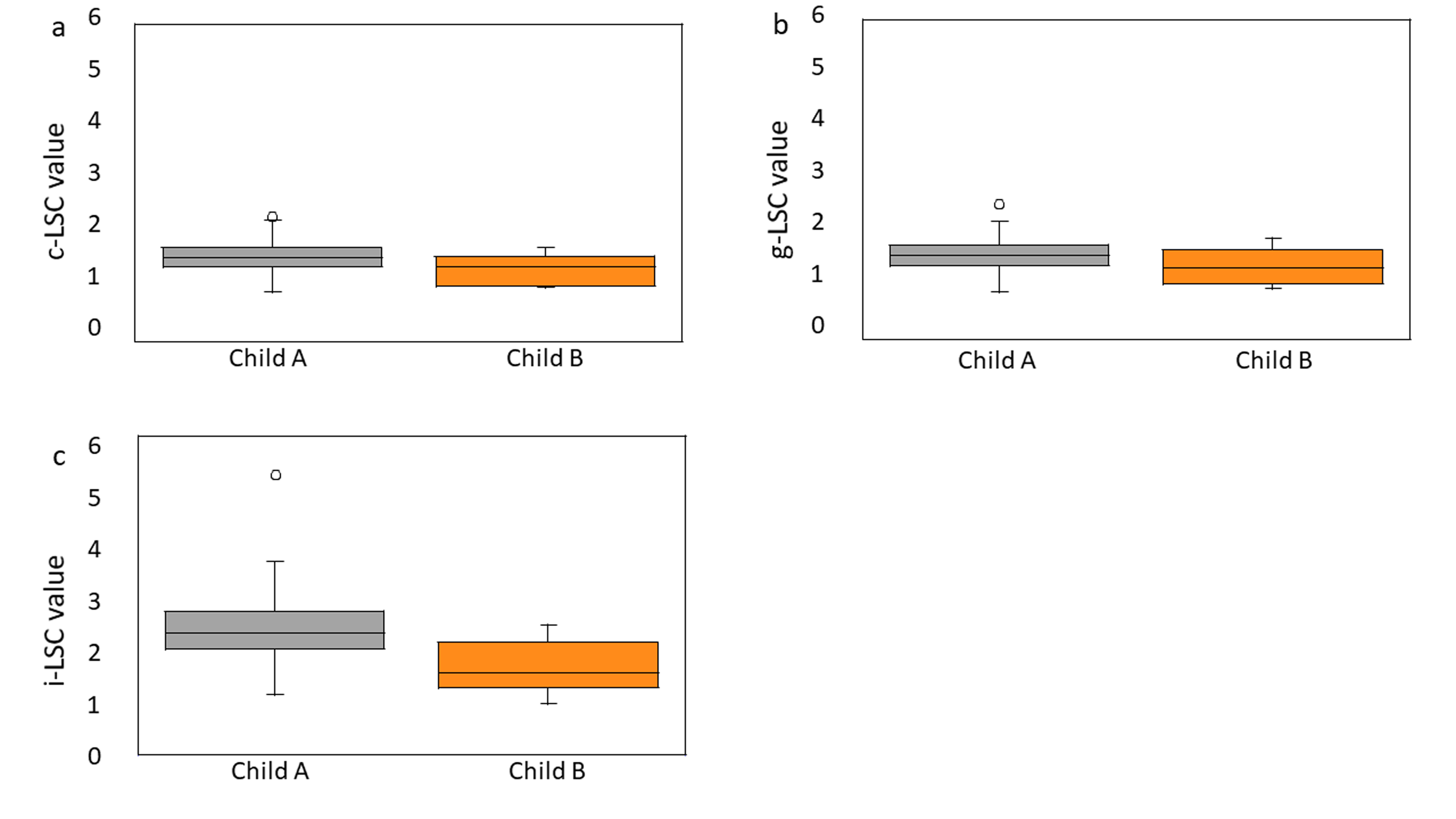

Supplement: Supplementary file 2 — Supplementary Figure 2 [file 261_2025_4817_MOESM2_ESM.tif]
