# Supplementary material for: Intracellular enhancement technique for gadoxetic acid-enhanced hepatobiliary-phase magnetic resonance imaging: evaluation of hepatic function
Source: Abdom Radiol (NY). 2025 Jan 31;50(8):3506–15. doi: 10.1007/s00261-025-04817-y (PMC12267321; doi:10.1007/s00261-025-04817-y)
Supplement: Supplementary file 3 — Supplementary Figure Legend [file 261_2025_4817_MOESM3_ESM.docx]

**Supplementary Figure 1**

The liver/spleen contrast obtained with each of the 3 scanning protocols.

1. all patients
2. patients with Child-Pugh class A
3. patients with Child-Pugh class B

**Supplementary Figure 2**

Comparison of the liver/spleen contrast (LSC) on HBP scans obtained with the 3 protocols in patients with Child-Pugh class A and B.

1. c-LSC
2. g-LSC
3. i-LSC

c-LSC, LSC at conventional hepatobiliary phase (HBP); g-LSC, LSC at HBP with g-denoising; i-LSC, LSC at HBP with g-denoising and the intracellular enhancement technique.
